# Parenting practices and trajectories of proactive coping assets among emerging adult Black men

**DOI:** 10.1002/ajcp.12758

**Published:** 2024-06-04

**Authors:** Christopher C. Collins, Elizabeth Kwon, Steven M. Kogan

**Affiliations:** ^1^ Human Development Family Science University of Georgia Athens Georgia USA; ^2^ Department of Public Health Baylor University Waco Texas USA

**Keywords:** adolescence, emerging adults, family, growth mixture model, health, positive youth development, rural Black men, substance use

## Abstract

Positive youth development (PYD) frameworks suggest that a critical response to investigating the challenges young Black men living in resource poor communities experience involves identifying contextual resources in young men's lives and personal assets that promote success. The following study examines heterogeneity in proactive coping assets trajectories, parental practices as predictors of developmental trajectories, and associated outcomes of each trajectory. The study sample consisted of Black emerging adult men living in rural Georgia (*N* = 504). At baseline, men were between the ages of 19 and 22 (*M*_age_ = 20.29; SD = 1.10). At wave four, the participants' mean age was 27.67 (SD = 1.39). Results of growth mixture modeling from waves 1 to 3 discerned three developmental trajectory classes of emerging adults' proactive coping assets: a *high and increasing* class (*n* = 247, 49%), a *low and stable* class (*n* = 212, 42%), and a *moderate and decreasing* class (*n* = 45, 9%). Trajectory classes were linked to baseline levels of parental support, coaching, and expectations. Analysis revealed that parental support and parental coaching predicted proactive coping asset trajectory class identification. Links were then investigated between emerging adults' proactive coping asset trajectory classes and wave four physical health, depression, and alcohol use. Results revealed significant associations between class identification, alcohol use, and physical health. Study findings provide evidence supporting the impact of parenting on emerging adult Black men, underscoring the need to expand resources that support parenting and emerging adult relationships.

## Highlights

- Study results revealed three proactive coping asset development trajectories.
- Parental practices of support and coaching were predictors of men's trajectory class identification.
- Substance use and physical health were significantly associated with trajectory class identification.

## INTRODUCTION

The transition from late adolescence to adulthood, termed emerging adulthood (~age 18–25), is a developmental phase that involves increasing independence from families and increased self‐reliance, decision‐making maturity, and accepting adult responsibilities (Arnett, 2000). Emerging adulthood represents a critical juncture that can affect development and success throughout an individual's life course. During this period, young people experience opportunities and vulnerabilities due to rapid transitions in their social roles and the experience of greater autonomy in their lives. Historically, within the United States, towns in the rural South have been marked by segregation with pervasive poverty and a dearth of community resources (Pathman et al., 2006). Rural communities in the South have the highest poverty rates (20%) compared with rural communities in other regions across the United States (Farrigan & Hertz, 2018). Experiences of individuals living in rural communities can include lower incomes, limited access to quality education, scarce employment opportunities, and a lack of healthcare resources (Matthews, 2017; Niccolai et al., 2022; Showalter et al., 2017). Rural communities with higher populations of Black residents experience more access disparities compared with other rural communities with a lesser percentage of Black residents (Kozhimannil & Henning‐Smith, 2019). Current research examining racial disparities suggests that rural Black populations experience higher mental health, social, and occupational challenges compared with their white counterparts (Breslau et al., 2005, 2006; Haynes et al., 2017). For the rural Black men who are the focus of the present study, limited educational and employment opportunities combined with racial discrimination can render the transition to productive emerging adult roles challenging (Bucknor, 2015; Shapiro et al., 2017). Many young rural Black men confront stressful environments that take a toll on their physical health, mental health, educational attainment, and economic success (Kogan et al., 2022; Probst & Ajmal, 2019).

Positive youth development (PYD) frameworks suggest that a critical response to investigating these challenges involves identifying resources in young men's lives and personal characteristics that promote development. Rather than approaching the challenges rural Black men experience from a deficit approach that highlights risk factors, PYD highlights cultivating intrapersonal assets and contextual resources associated with positive developmental outcomes (Gaylord‐Harden et al., 2018). This study focuses on how a contextual resource, parenting behavior, affects the development of proactive coping assets among young Black men. Building on adolescent research, we investigate how parenting behaviors affect young men's trajectories of proactive coping assets, including self‐regulation, future orientation, and vocational engagement. We then relate men's asset trajectories during emerging adulthood to their physical health, depression, and substance use.

A PYD approach underscores intrapersonal assets and the contextual resources that promote them. Among adolescents, intrapersonal assets include hopefulness, behavioral regulation, and school engagement (Lerner et al., 2005). These factors promote well‐being and reduce adolescent problem behaviors (Matthews et al., 2015). For example, Carvajal et al. (1998) linked optimism, hope, and self‐esteem to reductions in adolescent substance use. Jackman and Macphee (2017) found that future orientation deters impulsivity and engagement in delinquency and other risky behaviors. Among Black adolescents, future orientation has been associated with better mental health (So et al., 2016). Furthermore, previous research supports an association between motivational self‐regulation and pursuing career‐oriented goals (Shane & Heckhausen, 2016). However, few studies have examined the development of intrapersonal assets among emerging adults in general and young Black men in particular. Building on the adolescent literature, we consider a set of interrelated assets, which include self‐regulation, future orientation, and vocational engagement. Self‐regulation is intentionally managing one's conduct (Campos et al., 1989; Stormshak et al., 2018). Future orientation, a consciousness of the future self that influences behavior and decision making (Sharp et al., 2020), includes two aspects. Hopefulness refers to the extent to which individuals believe in their capacity to attain goals (C. R. Snyder et al., 1996), while perceived life chances refer to optimism regarding whether important life tasks can be accomplished (Beal & Crockett, 2010). Vocational engagement is an attitude and commitment toward occupational success (Lee et al., 2020).

Proactive coping theory provides a framework for understanding how interrelated intrapersonal assets converge onto one construct based on an underlying orientation. Prior literature conceptualizes proactive coping as a multidimensional construct of several intrapersonal assets (Greenglass et al., 1999). Aspinwall & Taylor (1997) posit that proactive coping is a self‐regulatory behavior in which individuals actively prepare for future stressors to avert potential adverse outcomes. Schwarzer and Taubert (2002) further expound upon this framework, asserting that proactive coping involves individuals envisioning their future goals and taking action‐oriented steps to achieve the envisioned success. Theoretical literature suggests that proactive coping is a process that manifests through several strategies, including the accumulation of resources, learning from past mistakes, learning from the experiences of others, and preparing for the future (Aspinwall & Taylor, 1997; Tian et al., 2023). Proactive coping differs from reactive coping in that while individuals recognize that future life stressors are inevitable, they focus on self‐regulation and goal management rather than a potential risk (Schwarzer & Luszczynska, 2008). This approach emphasizes resource and asset development rather than risk avoidance. Individuals with high proactive coping intentionally develop assets that create opportunities for personal growth while striving to improve their quality of life (Tylka & Wilcox, 2006).

Previous studies have explored intrapersonal asset development on health outcomes with children and adolescents (McClelland & Wanless, 2012; Urban et al., 2010). However, a better understanding of similar developmental processes during emerging adulthood is needed. Human developmental scholars have proposed that during emerging adulthood, individuals experience distinct neurological changes (Arnett, 2000). Scholars link intrapersonal assets to the development of the prefrontal cortex, which extends well into emerging adulthood (Jadhav & Boutrel, 2019). As a result of these neurological changes, proactive coping assets are likely to vary from year to year. Proactive coping assets should also increase with maturation in the prefrontal cortex. Research suggests a generally positive trend in emotion regulation from adolescence to adulthood (Zimmermann & Iwanski, 2014). Conversely, environmental challenges may undermine developmental competencies among emerging adults (Schulenberg et al., 2004; Urban et al., 2010). Therefore, emerging adulthood is a critical life course stage for long‐term health outcomes due to changes in intrapersonal asset development and contextual influences during the transition to adulthood (Sirois, 2015).

There may be heterogeneity in the trajectories of emerging adults' proactive coping assets. It is possible that subgroups of men will experience increases in assets, decreases in assets, or stability in assets across time. Prior studies have evaluated the heterogeneity in similar youth asset development, including intentional self‐regulation (Bowers et al., 2011) and social skill development (Oshri et al., 2017). Among emerging adults, prior studies have evaluated heterogeneity in deleterious trajectories, including depression (Yaroslavsky et al., 2013) and substance use (Huh et al., 2013). To our knowledge, no previous research has examined heterogeneity in young Black men's proactive coping assets over time. Similarly, few studies address the consequences of different trajectories of the development of proactive coping assets on well‐being. Consistent with research on asset development in adolescence and their associated outcomes later in life (Robson et al., 2020), we expect trajectories characterized by higher or increasing levels of proactive coping assets to forecast lower levels of depressive symptoms and alcohol use, and better health during emerging adulthood.

We consider an association between parenting behavior in late adolescence/early emerging adulthood and asset trajectories. Parenting behaviors are a powerful contextual resource associated with asset development during childhood and adolescence. Adolescents who receive high levels of discipline combined with warmth and involvement from their parents are likely to develop intrapersonal assets (Davis & Carlo, 2018). These assets include higher levels of self‐esteem and self‐regulation (Maholmes, 2018). The extent to which specific parenting practices are associated with emerging adult children outcomes is less clear. Parents typically adapt their parenting behavior during late adolescence and emerging adulthood to accommodate youths' increasing self‐reliance and autonomy (Nelson et al., 2011). Adaptations include relinquishing authority, supervision, and close control (Padilla‐Walker & Nelson, 2019). These adjustments support emerging adults' ability to develop autonomy and pursue opportunities associated with education, career exploration, and adult social relationships (Arnett, 2000). Parents who do not transition their parenting practices risk compromising autonomy, creating a sense of powerlessness, and undermining the development of essential skill sets of their emerging adult children (Cook, 2020).

Emerging research suggests that parenting practices supporting emerging adults continue to play an essential role in their development. Fosco et al. (2012) reported that emerging adults from families characterized by close, positive relationships exhibited improved control and regulation of emotions and behaviors. Liem et al. (2010), in their study of a cohort of high school seniors, found that authoritative parenting with older adolescents resulted in increased self‐worth and self‐efficacy during emerging adulthood. Past research with the present sample of rural Black men identified that parenting practices in the form of emotional support, vocational coaching, and high role expectations buffered the influence of racial discrimination on emerging adult Black men's alcohol use (Kogan & Bae, 2020). Other research finds that emerging adults with parents who maintain close, nurturing ties avoid heavy drinking (Madkour et al., 2017).

The current study investigates the association between Black men's exposure to parents' provision of social support, vocational coaching, high expectations for success, and proactive coping asset development during emerging adulthood. Previous studies link close positive family relationships to effortful control (Fosco et al., 2012) and reductions in emerging adult drinking (Madkour et al., 2017). Emerging adults benefit from instrumental forms of support that guide navigating work and education‐related pursuits (Kogan & Bae, 2020; Kogan & Brody, 2010). Particularly to Black families, theory and limited empirical research suggests that there are sex differences in how parents in Black families socialize their children. This research emphasizes the importance of parents maintaining high expectations for future success and communicating these expectations to their sons (Mandara et al., 2010).

## AIMS/HYPOTHESES

The current study has three specific aims. The first aim is to investigate heterogeneity in proactive coping asset trajectories among rural Black emerging adult men. Given the lack of previous studies, we make no specific hypotheses regarding the number or growth characteristics of trajectory classes that will emerge. The second aim is to link parental support, coaching, and high expectations in late adolescence/early emerging adulthood with proactive coping asset trajectory groups. In general, we expect parental support, parental coaching, and high parental expectations to link with trajectory classes characterized by higher mean levels and positive growth in proactive coping assets. Our third aim is to identify associations between trajectory classes and men's depressive symptoms, substance use, and physical health in young adulthood. We expect trajectories with increased proactive coping assets will forecast lower levels of depression and substance use and increased levels of self‐reported physical health. We controlled for parenting behavior during childhood to strengthen the inferences regarding the effects of emerging adult‐specific exposure to parenting practices on asset development. We also accounted for several potential confounders associated with proactive coping asset development, including maternal education, the presence of supportive mentors or romantic partners, living arrangements, and participant educational status.

## METHODS

### Participants

Study hypotheses were tested using African American Men's Health Project data. This longitudinal study explores risk and protective factors associated with young Black men's health outcomes. Participants included 504 men who self‐identified as Black or African American who resided in 1 of 11 rural counties in south Georgia. For data collection, research staff conducted in‐person visits to participants' homes or convenient, private locations in their community (e.g., private rooms in a local library). Participants completed audio computer assisted self‐interviews on a laptop computer. All study participants provided written informed consent.

Baseline (T1) data occurred between January 2012 and August 2013 (*N* = 504). The ages of study participants at baseline were 19–22 (*M*_age_ = 20.29; SD = 1.10). A second survey (T2) occurred between August 2013 and March 2015 (*N* = 422). The mean age at T2 was 21.84 (SD = 1.26). A third survey (T3) occurred between April 2015 and December 2016 (*N* = 407). The mean age of participants at T3 was 23.11 (SD = 1.25). A young adult follow‐up (T4) occurred between March 2019 and March 2021 when the participants' mean age was 27.67 (SD = 1.39). At T4, 351 (70%) of the initial sample provided data. We examined the link between retention and study variables at baseline. Retention at T4 was not associated with study variables at baseline. This suggests that data was missing at random.

Recruitment was conducted using respondent driving sampling (RDS). RDS is a preferred sampling method for recruiting hard‐to‐reach study populations, including young Black men (Kogan et al., 2011). RDS recruitment is designed to assess and attenuate the influence of bias in chain referral sampling and improve the approximation of a random sample for the targeted population. (Heckathorn, 2002). A concern of chain referral sampling involves sampling bias introduced by the seed participants. When RDS results in sufficiently long recruitment chains, the final sample becomes increasingly independent and not determinant of the initial seed participants. Additionally, RDS data collected about social networks allows for analysis to account for and control for the influence of network bias. These biases may be due to a participant's network size, recruitment efficacy, and the tendency to recruit only participants similar to oneself. In a previous study with the same sample (Kogan et al., 2016), analysis using RDS (Volz et al., 2007) revealed negligible biases in the study sample characteristics based on recruiter and seed participants' characteristics.

Initially, community liaisons recruited 45 seed participants. Upon completion of the survey, these participants were asked to refer three other potential study participants from their networks who met the inclusion criteria for the study (self‐reported Black men between the ages of 19 and 22 living in the targeted geographical area). Each participant was paid $100 after survey completion. Project staff followed up and communicated with referred individuals. Participants who initiated referrals received $25 per participant the study gained. Incentives for study participation were designed to show respect for participant's time and effort. We recognize that substantially high financial incentives can be considered coercive. We thus explored appropriate levels of financial incentives with community‐based focus groups and post‐data‐collecting briefings. We determined no concerns of coercion based on the study's financial incentives. The University's Institutional Review Board approved study protocols.

### COVID and study protocols

In March 2020, amid T4 data collection, the COVID pandemic began. By this time, data from 242 participants at T4 had been collected. Procedure adjustments allowed for data collection to occur remotely (*N* = 21). Study staff provided participants a link to complete the survey on personal devices, and no in‐person visits occurred. In October 2020, in‐person data collection resumed with adapted safety procedures, while the survey portion of data collection continued via study link. Under these adapted protocols, 88 participants provided data. A total of 351 participants provided T4 data. Modifications in study protocols were not associated with differences in alcohol use, depressive symptoms, or self‐reported health.

### Measures

#### Proactive coping assets

We assessed proactive coping assets with four scales obtained at T1, T2, and T3. Self‐regulation was measured with a 10‐item version of the Self‐Regulation Questionnaire (Brown et al., 1999). Example items include “I am able to accomplish goals I set for myself” and “When I am trying to change something, I pay attention to how I am doing.” The measure response scale ranged from 1 (*strongly disagree*) to 4 (*strongly agree*). Cronbach *α*'s were as follows: T1, *α* = .87; T2, *α* = .94; and T3, *α* = .96.

Two scales measured future orientation. (Sharp et al., 2020). The State Hope Scale (C. R. Snyder et al., 1996) is a six‐item measure. Example items included “If I should find myself in a jam, I could think of many ways to get out of it” and “I can think of many ways to reach my current goals.” The response scale ranged from 1 (*strongly disagree*) to 4 (*strongly agree)*. Cronbach *α*'s for each wave were as follows: T1 *α* = .85, T2 *α* = .90, and T3 *α* = .92. The Perceived Life Chances scale is a nine‐item measure. Men responded to whether they believed certain life goals were attainable. Example items include “You will have a job that pays well” and “You will be in good health most of the time.” The response scale ranged from 1 (*not sure at all*) to 4 (*very sure*). Cronbach *α*'s were as follows: T1, *α* = .90; T2, *α* = .92; and T3, *α* = .95.

Vocational engagement was measured using a 10‐item self‐report measure (Gore et al. (2007). Item examples included “I am a dependable employee” and “I often get in trouble at work” (reverse coded). The response scale ranged from 1 (*strongly disagree*) to 4 (*strongly agree*). Cronbach *α*'s for each wave were reported as follows, T1 *α* = .80, T2 *α* = .82, and T3 *α* = .80

#### Parenting practices

Parental support was measured at T1 using a six‐item Network Relationship Inventory (NRI) subscale. Men identify the parent that was most involved in their upbringing. If both parents raised men, they identified the parent with whom they had the closest relationship. Men most commonly selected their mothers. Example items include “How often do you turn to them for support with personal problems,” and “How often does this parent teach you how to do things that you do not know?” The measure response scale ranged from 0 (*neve*r) to 3 (*often*). Cronbach's *α* was reported as *α* = .94. Also, at T1, men completed a four‐item scale indexing parent's provision of vocational support (Kogan & Brody, 2010). Example items includes “Does this parent help you with your education or career plans?” The measure response scale ranged from 0 (*never*) to 3 (*very often*). Cronbach *α* was reported as *α* = .95. We measured parental expectations at T1 with a five‐item scale. (Kogan and Bae) Item examples included “This parent pushes me to take care of myself” and “This parent makes me take responsibility for my mistakes.” The items' response scale ranged from 1 (*strongly disagree*) to 4 (*strongly agree*). Cronbach *α* for the measure was *α* = .94.

#### Alcohol use

Alcohol use was measured at wave 4 using the Alcohol Use Disorder Identification Test, a 10‐item self‐report measure (Johnson et al., 2013). Men reported the frequency of and behaviors associated with their alcohol consumption. Item examples include “How many drinks containing alcohol do you have on a typical day when you are drinking,” and “Have you or someone else been injured as a result of your drinking?” Responses for items 1–3 ranged from 0 (*never*) to 4 (*4 or more times per week*). Responses for items 4–8 ranged from 0 (*never*) to 4 (*Daily or almost daily)*. Item responses for items 9 and 10 were 0 (*No*), 2 (*Yes, but not in the last year*), and 4 (*Yes, during the last year*). Men's responses were summed together for a total score. Cronbach *α* for the measure was *α* = .82.

#### Depressive symptoms

Men reported their depressive symptoms at wave 4 using the 20‐item Center for Epidemiologic Studies Depression Scale (CES‐D) (Radloff, 1977; Williams et al., 2007). Item examples included “How often did you have trouble keeping your mind on what you were doing,” and “How often did you think your life was a failure?” The response scale ranged from 0 (*rarely or none of the time [0‐1 days*]) to (*most or all of the time [6‐7 days])*. Cronbach's *α* for the measure was *α* = .86.

#### Physical health

Men self‐reported their general health at wave 4 with a 5‐item subscale from the Medical Outcomes Study Health Survey (Ware & Sherbourne, 1992). For item one, “How would you rate your overall health,” item responses ranged from 1 (*poor*) to 5 (*excellent*). For items two to five, the response scale ranged from 1 (*strongly disagree*) to 4 (*Strongly agree*). Item example included “I am as healthy as anybody I know.” Men's responses were standardized and then summed together for a total score. Cronbach's *α* for the measure was *α* = .70.

### Covariates

*Age*: Men reported their age at T1 in years (*M*_age_ = 20.29; SD = 1.10).

*Maternal Education*: Men reported the highest level of education their mothers completed. Responses ranged between 1 (*Grade 10 or below*) and 5 (*4‐year college degree or more*).

*Parenting during childhood*: At T2, men reported on their mothers' and fathers' parenting behaviors when they were growing up (before age 16; (Parker et al., 1997). Subscales on this measure include indifference, overcontrol, and abuse. Cronbach's *α* for the individual subscales ranged from 0.69 to 0.92. Subscales were strongly correlated (*r*'s = .47 ‐ .9; *ps *< .001) within and between parents. The subscales thus were summed within parents and then averaged between parents to create a childhood parenting variable. Higher scores meant exposure to higher levels of problematic parenting before emerging adulthood.

*Mentor support*: Men self‐reported if they had a mentor. A mentor was “a supportive adult (not a parent or romantic partner) at least 10 years older than you, who gives you support and advice” (1 = *Yes*, 0 = *No*).

*Romantic partner support*: Romantic Partner support was measured using a four‐item subscale from the Network Relationship Inventory (Furman & Buhrmester, 1985). The response scale ranged from 0 (*never)* to 3 (*very often*). Example items include “How often do you turn to them for support with personal problems?” and “How often do they help you figure out or fix things.” Cronbach's *α* was .73.

*Living arrangements*: Men responded to one item: “During most of the year, what are your living arrangements?” Response options included several living arrangements, such as “I live with my spouse or romantic partner” and “Live with friends or roommates my age.” Responses were recoded as a dichotomous variable to represent “I live with the family I grew up with” or “other.”

*Involvement in higher education*: Because of the age at baseline (19–22), some men may have completed their education while others were continuing or planning to continue their education. To index involvement in higher education, we created a dichotomous variable using information on educational attainment, current educational status (in higher education or not), and plans for entering higher education programs (including trade school or certifications). Men reported their educational attainment from 1 (*9th* *grade or lower*) to 8 (*Bachelor's Degree*). Men with no higher education responded to the question, “Do you plan to enroll in any kind of school or educational program in the next 6 months?” Men with no higher education and no plans to enter a program were coded as (0). Men with at least some higher education or intent to begin were coded as (1).

### Plan of analysis

Analyses were conducted using Mplus version 8.6 (Muthén, 1998). Mplus uses full‐information maximum likelihood (FIML) to account for missing data. Study participant retention at T4 was not associated with study variables at baseline. This suggests that data was missing at random, and FIML was appropriate. FIML provides estimates using all data available and does not drop any participant cases due to missing data. First, the proactive coping assets construct was developed. This construct consists of four latent indicators: self‐regulation, state hope, perceived life chances, and vocational engagement. Next, we conducted a longitudinal confirmatory factor analysis (LCFA) for the proactive coping assets construct to confirm its unidimensionality and invariance across time. We used marker variable scaling to set one factor variable's (Perceived Life Chances) loading to 1 and set the intercept to zero across time points. We used the following criteria to evaluate the model fit: comparative fit index (CFI) and Tucker‐Lewis index (TLI) ≥ 0.90 and root mean square error of approximation (RMSEA) ≤ 0.06. To test for measurement invariance, we compared additional models: weak/metric invariance (fixed lambdas across waves), strong/scalar invariance (fixed lambdas and intercepts across waves), and strict invariance (fixed lambdas, intercepts, and residual variance across waves). To compare nested models, we conducted the *χ* ^2^ difference test. This test evaluates the change in *χ* ^2^ and degrees of freedom Δ*χ* ^2^(*df*) between the nested models. If the *χ* ^2^ difference is considered nonsignificant, there is support for measurement invariance (Little, 2013; Satorra & Bentler, 2001).

Next, we used a growth mixture model (GMM) analysis, a linear model, to group individual proactive coping asset trajectories into classes. Model fit indices, including Akaike information criterion (AIC), Bayesian information criterion (BIC), and smallest class size percentage, were used to determine the optimal number of classes. Model fit for two, three, four, and five classes were assessed. The association between T1 parental support, parental coaching, parental expectations, and trajectory class membership was evaluated using the model‐based, three‐step approach (Asparouhov & Muthén, 2014). This three‐step approach includes identifying trajectory classes, including predictors, and then adding covariates into the model. We analyzed each predictor separately, along with identified covariates. We utilized the BCH (Bolck et al., 2004) approach to evaluate trajectory class effects on T4 alcohol use, physical health, and depressive symptoms. The BCH approach includes establishing model classes, identifying predictions of class membership, including covariates, and assessing the relationship between class membership and outcome variables (Bakk et al., 2013).

## RESULTS

Table 1 presents descriptive statistics of the study sample during the first wave of data collection. Of the men in the study, 256 (50.8%) were currently living with their family of origin, while 248 (49.2%) men had moved out of their parent's home. Only 145 men (28.8%) were involved or planned to be involved in higher education past their high school diploma/GED. Most men (242, 47.6%) reported their mother's highest level of education as a high school diploma/GED. In comparison, 90 men (17.7%) reported their mother had a 2‐year college degree, and 75 men (14.8%) reported their mother had a 4‐year degree or higher. Of the men in the study, 237 (47.2%) reported having some form of mentoring relationship with an older adult. Table 2 presents bivariate correlation along with means and standard deviations for proactive coping assets at each wave, predictor variables including covariates, and outcomes analyzed including alcohol use, physical health, and depression.

**Table 1:** Descriptive statistics *N* = 504.

Variable	*N*	Percentage
Living arrangement		
Living with family of origin	256	50.8
Other	248	49.2
Higher education involvement		
Yes	145	28.8
No	359	71.2
Maternal education		
10th grade or below	32	6.3
Grade 11	28	5.5
High school diploma or GED	242	47.6
2‐year college diploma/trade school	90	17.7
4‐year college degree or more	75	14.8
I don't know	35	6.9
Mentor support		
Yes	237	47.2
No	265	52.2

**Table 2:** Bivariate correlations.

Variable name	1	2	3	4	5	6	7	8	9	10	11	12	13	14	15	16
1. W1 Proactive coping assets	–															
2. W2 Proactive coping assets	0.51**	–														
3. W3 Proactive coping assets	0.37**	0.54**	–													
4. Parental support	0.27**	0.21**	0.22**	–												
5. Parental coaching	0.37**	0.28**	0.22**	0.61**	–											
6. Parental expectations	0.26**	0.20**	0.09	0.18**	0.23**	–										
7. W1 age	−0.16	−0.10*	−0.05	−0.16**	−0.22**	−0.03	–									
8. Maternal education	0.02	0.09*	0.09	0.07	0.09	0.05	−0.05	–								
9. Parenting during childhood	−0.05	−0.38**	−0.20**	−0.08	−0.10*	−0.07	−0.02	−0.01	–							
10. Mentor support	0.03	0.03	0.05	0.05	0.02	0.03	−0.01	−0.06	−0.04	–						
11. Romantic parnter support	0.14**	0.06	0.04	0.14**	0.135**	0.04	−0.04	0.01	−0.07	0.00	–					
12. Living arrangements	0.06	0.06	−0.08	0.11*	0.05	0.03	−0.17**	−0.10*	−0.10*	0.02	0.01	–				
13. W1 Educations	0.15**	0.21**	0.17**	0.09*	0.14**	0.10*	−0.03	0.13**	−0.03	−0.03	0.01	−0.08	–			
14. Alcohol use	−0.02	−0.11	−0.16**	0.02	0.02	0.02	−0.02	−0.02	0.08	−0.05	−0.04	−0.06	0.06	–		
15. Physical health	−0.28**	−0.24**	−0.25**	0.00	−0.08	−0.04	−0.01	0.00	0.06	0.05	0.00	−0.01	−0.10	0.12*	–	
16. Depression	−0.14**	−0.21**	−0.36**	−0.10	−0.13*	−0.11*	0.03	−0.11*	0.22**	−0.01	−0.01	−0.04	−0.10	0.22**	0.18**	
Means	113.57	113.53	108.71	11.58	8.40	17.28	20.26	3.50	5.71	0.47	5.05	0.49	0.29	4.24	0.00	14.82
Standard deviation	11.74	14.11	17.55	5.19	3.59	3.48	1.08	1.21	7.64	0.50	4.01	0.50	0.45	5.14	3.29	9.18

*Note*: Means and standard deviations (*N* = 504).

W1, W2, W3 = Wave 1, 2, and 3, respectively.

* *p* < .05

** *p* < .01.

### Longitudinal confirmatory factor analysis

At each wave, proactive coping assets indicators loaded on a single factor (*λ*s > 0.30 *p* < .05) and in the expected direction. This model fit data is reported as follows: *χ* ^2^(*39*) = 134.106, *p* < .000; CFI = 0.96, TLI = 0.93, RMSEA = 0.06 (90% CI 0.053, 0.079), and SRMR = 0.09. We then tested a series of nested models investigating invariance across time. To test for weak/metric invariance, we compared the configural (unconstrained) model to a model with lambdas fixed across each wave (Little, 2013). The model fit for the weak/metric invariance is reported as follows: *χ* ^2^(*45*) = 144.942, *p* < .000; CFI = 0.95, TLI = 0.93, RMSEA = 0.06 (90% CI 0.054, 0.079), and SRMR = 0.10. No statistical difference was found between these two models Δ*χ* ^2^(*6*) = 10.836, ΔCFI = 0.01, ΔRMSEA = 0.00 ΔSRMR = 0.01. Next we compared the weak/metric invariance model with a model that had fixed lambdas and intercepts across each wave to test for strong/scalar invariance. Model fit for the strong invariance model is reported as follows: *χ* ^2^(*51*) = 155.839, *p* < .000; CFI = 0.95, TLI = 0.93, RMSEA = 0.06 (90% CI 0.053, 0.075), SRMR = 0.11. No statistical difference was found between the two models Δ*χ* ^2^(*6*) = 10.897, ΔCFI = 0.00, ΔRMSEA = 0.00, ΔSRMR = 0.01. Finally we compared the strong invariance model to a model with fixed lambdas, intercepts and residual variance across each wave to test for strict invariance. Model fit for the strict invariance model is reported as follows: *χ* ^2^(*59*) = 170.665, *p* < .000; CFI = 0.94, TLI = 0.94, RMSEA = 0.06 (90% CI 0.051, 0.072), and SRMR = 0.12. No statistical difference was found between the two models Δ*χ* ^2^(*8*) = 14.826, ΔCFI = 0.01, ΔRMSEA = 0.00, ΔSRMR = 0.01. LCFA findings, support the unidimensionality and invariance across time of the proactive coping assets construct.

### Trajectory classes

**Figure 1:**
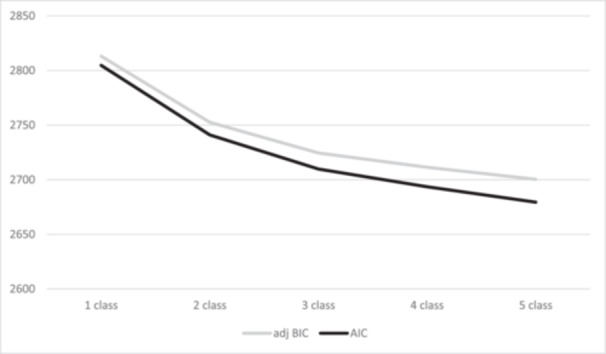
Elbow plot of the adjusted Bayesian Information Criterion and Akaike Information Criterion.

**Figure 2:**
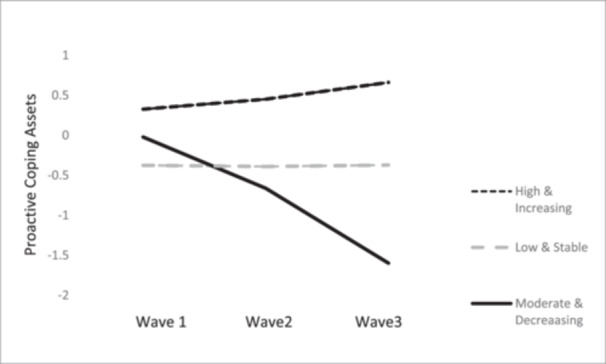
Three‐class solution of linear growth mixture model.

Table 3 provides model fit indices for the growth mixture model selection in a number of classes. Model fit indices suggested that adding more classes continuously increases the model fit. Three class model was selected based on inspection of elbow plots (see Figure 1) of AIC and adjusted BIC as well as model parsimony, interpretability of the results, and sample size of the smallest group. Although smaller AIC and adjusted BIC values indicate better model fit, minimal changes in these values mean that estimating more classes does not add gains. The three‐class model evidenced discontinuous changes in the AIC and the BIC. The three‐class model's smallest class also had a reasonable percentage (9%) of the sample for examining class differences. The three classes are presented in Figure 2. Individuals in the *high and increasing* class (*n* = 247, 49%) evidenced high starting points and positive growth across time. Individuals in the *low and stable* class (*n* = 212, 42%) evinced the lowest starting point, and their levels remained stable over time. The final class, *moderate and decreasing* (*n* = 45, 9%), evidenced moderate proactive coping assets at T1. However, this class experienced a decrease across time points, resulting in much lower proactive coping asset scores at T3 compared with the other classes.

**Table 3:** Model fit for linear growth mixture model models for proactive coping assets.

Classes size	Log likelihood	AIC	Adj BIC	SC *N* (%)	BLRT *p* value
1	−1394.351	2804.702	2813.09	–	–
2	−1359.525	2741.05	2752.583	55 (0.11)	<0.0001
**3**	**−1340.901**	**2709.801**	**2724.48**	**45 (0.09)**	**<0.0001**
4	−1329.764	2693.528	2711.352	9 (0.02)	<0.0001
5	−1319.707	2679.415	2700.385	14 (0.03)	<0.0001

*Note*: Chosen class is shown in bold.

Abbreviations: Adj BIC, sample size adjusted BIC; AIC, Akaike information criterion; BIC, Bayesian information criteria; BLRT, Bootstrap Likelihood Ratio Test for k‐1 (H0) versus k classes; SC, smallest class size.

### Predictors and outcomes

Table 4 presents the links between each parenting variable at T1 and class membership. Each predictor, parental support, coaching, and expectations, was analyzed in a separate model along with identified covariates. Individuals who reported higher levels of parental support had significantly higher odds of being in the *high and increasing* class compared with the *moderate and decreasing* class (AOR 1.16, *p* < .004) or the *low and stable* class (AOR 1.12, *p* < .001). Similarly, individuals who reported higher levels of parental coaching also had significantly higher odds of being in the *high and increasing* class rather than the *moderate and decreasing* class (AOR 1.17, *p* < .033) or the *low and stable* class (AOR 1.22, *p* < .002). Parental expectations were not a significant predictor of trajectory class.

**Table 4:** Multinomial logistic regression OR and CIs for predictors (*N* = 504).

Predictors model	High and increasing versus, Moderate and decreasing				High and increasing versus Low and stable				Moderate and decreasing versus, Low and stable			
	Mhi	Mmd	AORs	95% CIs	Mhi	Mls	AORs	95% CIs	Mmd	Mls	AORs	95% CIs
Parental support model	12.70	10.10	1.16	[1.05, 1.29]**	12.70	10.6	1.12	[1.05, 1.20]***	10.10	10.6	0.97	[0.88, 1.07]
Age at W1	20.10	20.50	0.67	[0.44, 1.01]	20.10	20.4	0.82	[0.58, 1.14]	20.50	20.4	1.22	[0.80, 1.87]
Maternal education	3.51	3.20	0.99	[0.67, 1.47]	3.51	3.55	1.02	[0.76, 1.36]	3.20	3.55	1.02	[0.66, 1.58]
Parenting during childhood	4.73	10.11	0.87	[0.82, 0.93]***	4.73	5.94	0.94	[0.89, 1.00]*	10.11	5.94	1.08	1.02, 1.15]**
Mentor support	0.50	0.43	1.01	[0.41, 2.48]	0.50	0.44	1.47	[0.77, 2.79]	0.43	0.44	1.46	[0.56, 3.79]
Romantic partner support	7.79	6.59	0.94	[0.84, 1.06]	7.79	6.16	1.05	[0.96, 1.14]	6.59	6.16	1.11	[0.99, 1.25]
Living arrangement	0.52	0.52	0.28	[0.10, 0.78]*	0.52	0.48	1.03	[0.54, 1.99]	0.52	0.48	3.66	1.28, 10.53]*
W1 Higher Education	0.35	0.14	5.17	[1.61, 16.56]**	0.35	0.25	2.02	[0.93, 4.43]	0.14	0.25	0.39	[0.11, 1.35]
Parental coaching model	9.22	7.55	1.17	[1.01, 1.34]*	9.22	7.64	1.22	[1.07, 1.38]**	7.55	7.64	1.05	[0.93, 1.18]
Age at W1	20.10	20.50	0.68	[0.46, 1.01]	20.10	20.40	0.86	[0.61, 1.20]	20.50	20.40	1.26	[0.83, 1.93]
Maternal education	3.51	3.20	1.01	[0.69, 1.48]	3.51	3.55	1.00	[0.74, 1.34]	3.20	3.55	0.99	[0.63, 1.55]
Parenting during childhood	4.73	10.11	0.88	[0.82, 0.94]***	4.73	5.94	0.94	[0.89, 1.00]	10.11	5.94	1.08	1.02, 1.14]**
Mentor support	0.50	0.43	1.06	[0.43, 2.58]	0.50	0.44	1.56	[0.81, 3.00]	0.43	0.44	1.47	[0.56, 3.83]
Romantic partner support	7.79	6.59	0.95	[0.85, 1.07]	7.79	6.16	1.04	[0.95, 1.14]	6.59	6.16	1.09	[0.98, 1.22]
Living arrangement	0.52	0.52	0.32	[0.12, 0.89]*	0.52	0.48	1.13	[0.57, 2.21]	0.52	0.48	3.47	[1.20, 10.06]*
W1 Higher Education	0.35	0.14	4.38	[1.35, 14.21]**	0.35	0.25	1.70	[0.78, 3.71]	0.14	0.25	0.39	[0.11, 1.39]
Parental expectations	17.64	17.24	1.07	[0.89, 1.29]	17.24	16.86	1.13	[0.92, 1.39]	17.24	16.86	1.05	[0.92, 1.21]
Age at W1	20.10	20.50	0.62	[0.42, 0.91]	20.10	20.40	0.76	[0.55, 1.04]	20.50	20.40	1.23	[0.81, 1.85]
Maternal education	3.51	3.20	1.04	[0.71, 1.55]	3.51	3.55	1.05	[0.79, 1.40]	3.20	3.55	1.00	[0.64, 1.56]
Parenting during childhood	4.73	10.11	0.88	[0.82, 0.93]***	4.73	5.94	0.94	[0.90, 0.99]*	10.11	5.94	1.08	1.02, 1.14]**
Mentor support	0.50	0.43	1.11	[0.46, 2.67]	0.50	0.44	1.56	[0.83, 2.96]	0.43	0.44	1.41	[0.55, 3.60]
Romantic partner support	7.79	6.59	0.97	[0.87, 1.08]	7.79	6.16	1.06	[0.98, 1.14]	6.59	6.16	1.09	[0.98, 1.22]
Living arrangement	0.61	0.52	0.33	[0.12, 0.91]	0.61	0.48	1.09	[0.56, 2.13]	0.52	0.48	3.35	1.13, 9.93]*
W1 Higher education	0.35	0.14	4.60	[1.46, 14.49]**	0.35	0.25	1.79	[0.86, 3.73]	0.14	0.25	0.39	[0.11, 1.36]

Abbreviations: AOR, adjusted odds ratio; CI, confidence interval; Mls, mean score of the low stable group; Mmd, mean score of the moderate decreasing group.

* *p* < .05

** *p* < .01

*** *p* < .001.

Outcomes associated with trajectory class are presented in Table 5. Alcohol use was significantly associated with *the moderate and decreasing* class compared with the *high and increasing* class (*p* < .012) and with the *moderate and decreasing* class compared with the *low and stable* class (*p* < .002). Physical health was higher in the *high and increasing* classes than in the *low and stable* classes (*p* < .017). Depressive symptoms were not significantly associated with trajectory class.

**Table 5:** Means, estimates, and standard errors for W4 outcomes.

Predictors variable	High and increasing versus, Moderate and decreasing				High and increasing versus Low and stable				Moderate and decreasing versus, Low and stable			
	Mhi	Mmd	Est.	SE	Mhi	Mls	Est.	SE	Mmd	Mls	Est.	SE
Depression	16.91	26.99	6.76	33.16	16.91	16.22	−3.85	27.95	26.99	16.22	10.61	40.37
Alcohol use	15.28	77.07	61.72**	24.61	15.28	−12.86	−30.76	17.01	77.07	−12.86	92.47**	29.26
Physical health	11.26	6.55	−2.93	11.25	11.26	−14.93	−23.65*	11.25	6.55	−14.93	20.73	13.07

*Note*: *M* = Intercept for high increasing (Mhi), moderate decreasing (Mmd), and low stable (Mls).

Age, parental education, and living with parent, were controlled for depression, alcohol use, and physical health.

Wave one scores were controlled for *p < .05, **p < .01.

## DISCUSSION

A PYD framework informed this study. This approach aims to identify intrapersonal assets and contextual resources and how accessing these resources can affect development (Guerra & Bradshaw, 2008). Specifically, we examined heterogeneity in trajectories of emerging adults' proactive coping assets, the potential for parenting practices to forecast trajectory group membership, and the association of trajectory groups on young adult well‐being. Study findings revealed heterogeneity in men's proactive coping assets over time. Three trajectory classes emerged: *high and increasing*, *low and stable*, and *moderate and decreasing*. Parental support and parental coaching were associated with trajectory class identification. Men who reported higher levels of parental support and coaching were likelier to be in the *high and increasing* trajectory class than the *low and stable* or the *moderate and decreasing* classes. Men within the *high and increasing* class evidenced significantly less alcohol consumption than the *moderate and decreasing* class and better self‐reported physical health than the *low and stable* class.

Finding multiple trajectory classes is consistent with previous research demonstrating heterogeneity in emerging adult developmental assets including self‐control (Burt et al., 2014) and emotion regulation (Zimmermann & Iwanski, 2014). Considerable neurodevelopment occurs during the emerging adult years. Maturation in the prefrontal cortex occurs well into the individuals' mid‐20s (Griffin et al., 2015; O'Connor et al., 2009). Prior theoretical and empirical literature also underscores the emerging adult years as a time of considerable variability in social roles, which, combined with continued brain development, highlights the potential for different developmental pathways to emerge (Schulenberg et al., 2004). A discussion of each trajectory class, class predictors, and class outcomes follows.

### High and increasing class

Despite men growing up in underresourced, rural communities, nearly half of the men (49%) experienced high starting points and positive growth in proactive coping assets. These findings are consistent with past research suggesting that positive growth in self‐regulatory processes occurs during emerging adulthood for most young people. A previous study reported higher growth in emotional regulation during emerging adulthood compared with adolescence (Zimmerman et al., 2013). Current study findings indicated that men in the *high and increasing* group were likelier to experience supportive parenting and vocational coaching at baseline. Past research has linked similar parenting practices to developmental assets among adolescents (Baker & Hoerger, 2012) and emerging adults (Augustine et al., 2022). Specifically, high‐quality parent–youth relationships promote emerging adult self‐regulation development (Shannon et al., 2016). Controlling for early parenting practices that may have an association with development during emerging adulthood potentially strengthens our conclusions that the parenting men received as late adolescents/emerging adults (and not at earlier developmental stages) made a unique difference in their ongoing development.

Men in this trajectory group also reported better physical health and less alcohol use. These findings are consistent with previous research among youth linking the development of self‐regulatory processes in earlier life stages with improved physical health (Howard & Williams, 2018) and linking future orientation to reduced substance use (Robbins & Bryan, 2004). Our research extends these findings to associations with proactive coping assets and underscores how continued development during this stage may support future well‐being.

### Low and stable class

A second trajectory class characterized 42% of the men in this sample. These men reported low levels of proactive coping assets at baseline, and their asset levels remained stable over time. Transitioning out of high school, these men experienced lower levels of hopefulness for the future and were unlikely to set goals toward future educational attainment compared with the *high and increasing* class. Importantly, these men were less likely to report involvement in postsecondary education or training. Research documents multiple transitional challenges experienced by individuals who do not seek higher education or postsecondary training (Rosenbaum et al., 2015). Noncollege bound individuals experience less financial growth and stability (Mitchell & Syed, 2015), which can impact their emerging adult experiences. Living in racially segregated rural communities also restricts Black men's exposure to diverse career opportunities. This lack of exposure may make individuals less likely to pursue postsecondary education or training (Kerpelman & Mosher, 2004).

Men's *low and stable* asset developmental trajectory may also reflect challenges associated with growing up in and transitioning to adulthood in the rural South. For Black families, poverty in rural areas is endemic (Wimberley & Morris, 2002). Their poverty status reflects the dominance of a low‐wage workforce, resource‐intensive industries, and the lack of opportunities in rural communities previously addressed. Families with little discretionary income residing in rural areas can have limited access to public resources (including transportation and recreational facilities) and difficulties accessing physical and mental healthcare (Probst & Ajmal, 2019; Tickamyer, 2006). Stress associated with limited family resources can contribute to more chaotic living conditions during childhood and adolescence, including disorganization, instability, and unpredictability (Maholmes & King, 2012), which can undermine asset development (Herd et al., 2020). Consistent with this background, men in the *low and stable* class experienced significantly less parental support and coaching than the *high and increasing* class in emerging adulthood. They also reported elevated levels of adverse parenting exposures during childhood. These men may have lacked critical family support for envisioning beneficial future life paths and entered the transition to adulthood with low expectations for educational and career opportunities. For men within the *low and stable* class, limited employment opportunities and cumulative stress associated with family processes may have undermined emerging adults' proactive coping asset development.

Men in the *low and stable* class also evinced the lowest self‐reported health among the groups. This finding is concerning, given disparities in a wide range of downstream health outcomes for rural Black men. These health disparities are directly linked to growing up in socioeconomically challenging rural communities (Brody et al., 2013; Shonkoff et al., 2009). Furthermore, Black men disproportionately experience higher levels of chronic health challenges, including diabetes, cancer, and heart disease (Gilbert et al., 2016). These health challenges underscore the importance of identifying contextual resources supporting rural Black men's health.

### Moderate and decreasing class

A modest percentage of the men (9%) exhibited trajectories that were particularly concerning. These men began emerging adulthood with modest levels of proactive coping assets but experienced declines over time. By Wave 3, these men showed the lowest levels of proactive coping assets among the three classes. Identifying this trajectory is consistent with the proposition that emerging adulthood is a sensitive period when contextual influences may undermine development (Schulenberg et al., 2004). These men were less likely to report parental support and vocational coaching than the *high and increasing* group. Studies suggest that parental support is a critical protective factor for adolescents living in underresourced communities (Clark et al., 2011). Though these men transitioned to adulthood with moderate levels of proactive coping assets, they may have struggled with their new social roles and lacked the support needed to navigate this transition.

Examining study covariates may provide further insight into the experience of men in the *moderate and decreasing* class. These men were less likely to report education or training past high school. Growing up in underresourced, rural communities affects emerging adults' educational aspirations (Cox et al., 2014). It may be that these men's social context, with limited parental support and coaching, led them to self‐select from additional educational opportunities. These men were also more likely to continue living in their childhood homes than men in the *high and increasing class*. This finding may be the result of difficulties in managing transition‐related challenges (finding work, attending training, or attending higher education programs). Prior studies also suggest that continuing to live in their parent's home may inhibit development associated with independence and autonomy (Kins et al., 2009; Scales et al., 2016). This living arrangement could have had a similar impact on these emerging adults' asset development.

Examination of covariates further indicated that these men were exposed to increased levels of problematic parenting behaviors while growing up, including parental indifference, overcontrolling, and abuse. Such adverse parenting practices affect multiple domains of development (Repetti et al., 2002) and can have lasting implications for downstream development (McMahon, 2014). In the context of an underresourced community, the negative effects of adverse parenting are often amplified (Berzin & De Marco, 2010).

Men in the *moderate and decreasing* class reported elevated alcohol use. This finding is consistent with past research linking negative parental behaviors to emerging adult substance use (S. M. Snyder & Merritt, 2016). Particularly for youth exposed to adverse experiences, a lack of positive assets may increase problematic alcohol use (Shin et al., 2019). These men, living in low resource environments with little parental support, may have struggled to adapt to emerging adulthood's changing roles. These challenges may have resulted in men's higher levels of substance use compared with the *high and increasing class*.

### Limitations

Limitations to this study are noteworthy. Conceptually, proactive coping is multidimensional on several levels. The current study explores three factors, self‐regulation, future orientation, and vocational engagement, that converge into our proactive coping construct. Other studies exploring proactive coping have identified as many as seven factors in a construct (Greenglass et al., 1999). Variation exists in current self‐regulation and intrapersonal asset research. These dynamic human developmental processes reflect adaptive complex systems that scientific data collection may not fully encompass (Nigg, 2017). We recognize that other underlying developmental processes may influence the associations between parenting behaviors, young Black men's proactive coping development, and well‐being. Particularly to ethnic‐racial adolescents, researchers have recently explored associations between ethnic‐racial socialization, proactive coping assets, and experiences of discrimination (DeLapp & Williams, 2021; McDermott et al., 2018; Salcido & Stein, 2024). Future studies may explore these associations and their impact on young Black men's development.

The current study sample consisted of emerging adult Black men from rural Georgia. Study findings may not apply to other populations including but not limited to individuals of varied ethnic racial identities, women, and nonrural young Black men. There are contextual factors that may influence these men's health outcomes that were not measured within the current study. Additionally, study measures relied upon participant self‐report, subject to individual response recall and social desirability bias (Larson, 2019). These limitations notwithstanding, the present study provides empirical evidence on the relationship between parenting practices and emerging adult proactive coping asset development and associated outcomes.

### Implications

Study findings suggest that parenting behaviors can promote the development of proactive coping assets associated with better physical health and less substance use during emerging adulthood. Results from this study can inform policy and programs designed to support men during this critical transition. Parental training programs, informed by behavioral, cognitive, and social learning perspectives, have proven efficacious in enhancing parental skills and practices (Chan et al., 2022; Nowak & Heinrichs, 2008; Wilson et al., 2016). However, these programs are typically designed for parents of younger children and adolescents. Few programs at present address the needs of Black emerging adults. A notable exception is the Adults in the Making (AIM) intervention, which explicitly targets Black families with high school seniors from underresourced communities and the development of their family processes and self‐regulation of youth (Brody et al., 2010). Current study results support the relevancy of expanding similar programs and resources that strengthen parents' ability to support their emerging adult children.

## CONCLUSION

The current study investigates the association between Black men's exposure to parents' provision of social support, vocational coaching, high expectations for success, and proactive coping asset trajectory development during emerging adulthood. We also aimed to identify outcomes (depression, physical health, and alcohol use) associated with asset developmental trajectories. Three distinct trajectory classes were identified: *high and increasing*, *moderate and decreasing*, and *low and stable*. Study findings suggest that parents who provide support and coaching during emerging adulthood may influence young Black men's growth in proactive coping assets. Additionally, young Black men who continuously develop proactive coping assets during emerging adulthood may experience better physical health and lower alcohol use. These findings suggest that while parents need to adjust their parenting behaviors and relinquish control toward their adult children to allow for autonomy and self‐reliance, parental involvement may still influence emerging adult well‐being.

### AUTHOR CONTRIBUTIONS

Christopher C. Collins conceived of the study and wrote the first draft of the manuscript. Elizabeth Kwon assisted with the statistical analysis of the study. Steven M. Kogan assisted with study conception, provided the study data, contributed to the writing, and commented on drafts of the manuscript.
